# Reproductive Outcomes of Infertile Males With Novel Genetic Defects

**DOI:** 10.7759/cureus.63139

**Published:** 2024-06-25

**Authors:** Huda M Omran, Mohammed S Almaliki

**Affiliations:** 1 Genetics: Molecular Genetics, Pulse Health Training Center, Al Jenan Medical Center, Manama, BHR; 2 Pediatrics, American Mission Hospital, Manama, BHR

**Keywords:** male, infertility, karyotyping, cftr gene, dna sequencing

## Abstract

Various chromosomal structural aberrations and genetic mutations have been discovered in infertile couples. Some have no obvious loss of genetic material; they are usually phenotypically normal people with reproductive issues. Males with these illnesses may have infertility and abnormal sperm analysis. However, positive sperm have also been detected in the ejaculation of some patients. As a result, knowing about these problems and how common they are can influence the fertility treatment that couples receive to achieve pregnancy and the birth of healthy newborns.

## Introduction

Men are responsible for 50% of infertility problems in couples, and up to 7% of men are estimated to be infertile [1]. In approximately 50% of cases of male infertility, which is known as idiopathic infertility, the fundamental cause is still unknown despite significant advancements in our knowledge of human reproductive physiology [2]. Given that there are most likely thousands of genes involved in human spermatogenesis, most idiopathic instances are most likely genetic in origin. From the treatment perspective, only some of the genes associated with spermatogenesis, testis descent, and testis determination are typically important. The androgen receptor (AR) gene [3], whose mutations cause androgen insensitivity syndrome and spermatogenic damage, and the CFTR gene, whose mutations cause cystic fibrosis and loss of vas deferens [4], both play important roles in gonadal differentiation. However chromosomal defects are become of increasing importance in male infertility.

This retrospective case series was reviewed and approved by the Institutional Review Board (IRB) of Jenan Medical Center. The researchers examined the medical records of 36 male patients attending a genetic clinic at the Center for Male Factor Infertility. Clinical history, family history, semen analysis, hormonal profile, and genetic analyses were collected. Patients with non-genetic causes of infertility (e.g., sexually transmitted infections or varicocele) were excluded from this study. Informed consent was obtained from all patients described in this report to publish their clinical details and any accompanying images. Out of this group, a geneticist identified three cases that were potentially carrying a genetic abnormality, though these cases were not consecutive.

## Case presentation

Patient 1

A 35-year-old man was referred to a fertility clinic in November 2022 due to non-obstructive azoospermia and low testosterone. He underwent genetic testing and was found to be a carrier of the cystic fibrosis (CF) gene mutation. The patient was heterozygous for CFTR (c.3209G>A). In addition, he had another mutation in the UBR2 gene (heterozygous for UBR2 c.1674+5G>T). The patient and his partner were counseled about their options for fertility treatment, which included in vitro fertilization (IVF) with preimplantation genetic testing (PGT) to avoid passing on the gene mutations to their offspring.

Patient 2

**Figure 1:**
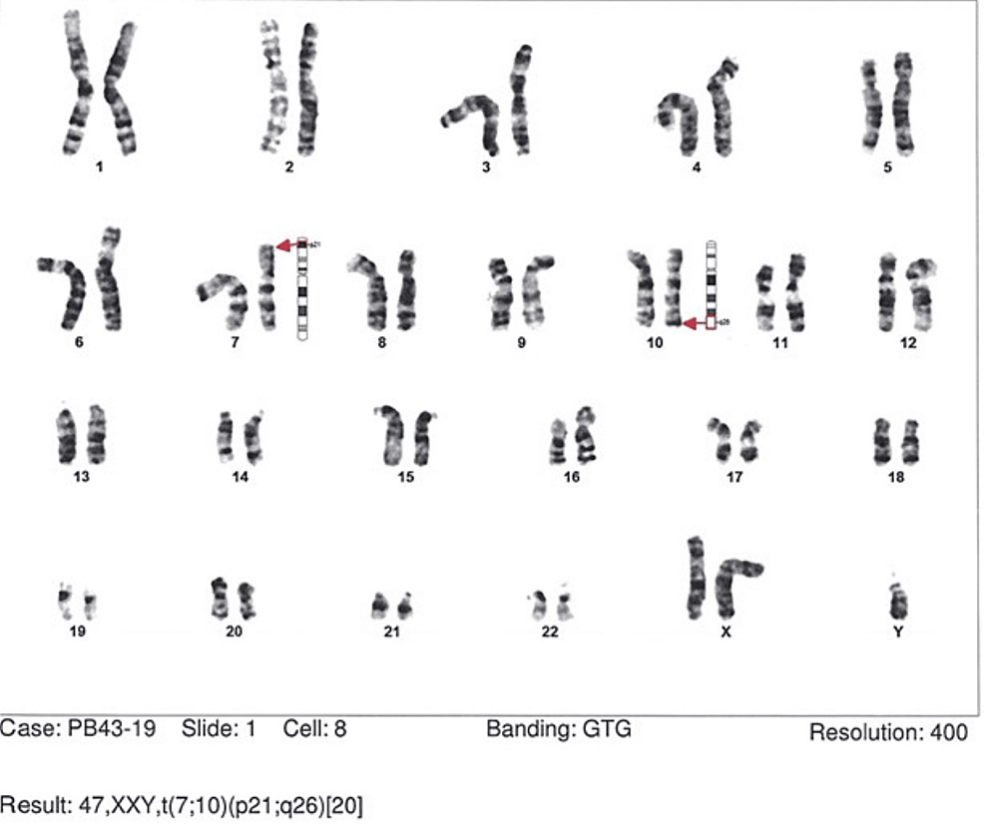
Translocation between chromosomes 7 and 10, 47XXY, t (7;10) (p21; q26)

A 24-year-old man presented to the genetic clinic in March 2023 with a history of infertility due to non-obstructive azoospermia. High levels of follicle-stimulating hormone (FSH) and luteinizing hormone (LH) and low levels of testosterone and mean testicular volumes were observed. He was diagnosed with Klinefelter syndrome, a genetic condition that affects males and is caused by an extra X chromosome, with a translocation between chromosomes 7 and 10, 47XXY,t(7;10) (p21;q26) as shown in Figure 1.

The patient also had Y chromosome changes and had partial duplication of the AZFc region(b2/b3). Similar variation was reported in his father who had normal fertility. Therefore, the direct correlation of this Y chromosome change to his clinical condition was excluded. Klinefelter syndrome is associated with testicular failure, which can result in infertility due to a lack of sperm production [5]. The patient was counseled about his options for fertility treatment, for example, testicular sperm extraction (TESE).

Patient 3

**Figure 2:**
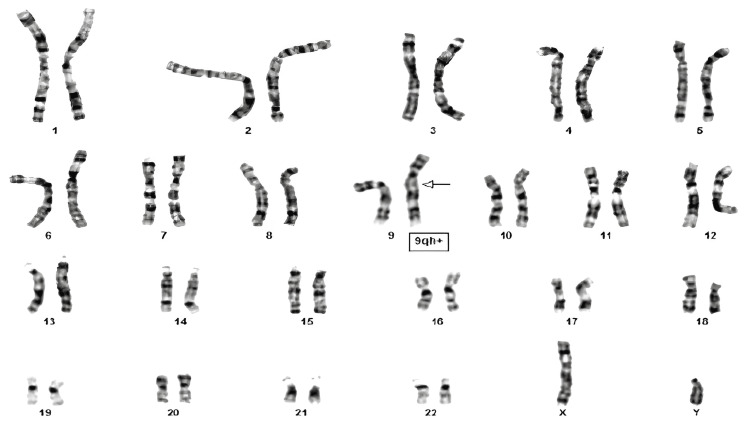
Chromosome 9 with increased length of heterochromatin 46XY, 9qh+

A 35-year-old male presented to a fertility clinic in January 2022 with a history of infertility. He had normal hormonal levels, but a testicular biopsy revealed germ cell maturation arrest (Johnson score 8/10). He reported having regular sexual intercourse with his partner for the past two years without any success in achieving pregnancy. The patient had previously undergone a semen analysis, which revealed oligoasthenozoospermia. Further evaluation with a chromosomal analysis showed chromosome 9 with an increased length of heterochromatin 46XY, 9qh+ as shown in Figure 2.

However, no other genetic abnormalities had been detected with DNA sequencing. The patient was counseled on the potential risks and options available for assisted reproductive technology (ART). In vitro fertilization (IVF) with preimplantation genetic testing (PGT) was recommended to reduce the risk of passing on the chromosomal abnormality to offspring.

The patient and his partner underwent IVF with PGT after one year of hormonal therapy, resulting in the selection of a normal embryo for transfer. The couple achieved a successful pregnancy and delivered a healthy baby without complications.

## Discussion

Human infertility may have a hereditary component, although the precise defects implicated and the genetic process by which they are transmitted remain unclear [6,7]. The present report aimed to present three cases of male patients who consulted the genetic unit because of infertility problems. A range of karyotype findings, from normal karyotyping to chromosomal abnormality with Y chromosome alterations, were discovered by cytogenetic analysis employing the GTG-banding approach. Cytogenetic investigations are vital for the identification of many different disorders, even though they can occasionally be difficult. Furthermore, fluorescence in situ hybridization (FISH) and further additional molecular techniques like Exome sequencing can enhance the efficacy of cytogenetic techniques in achieving a clinical diagnosis [8]. When combined with traditional karyotyping, the latter can significantly overcome the shortcomings of traditional bands in terms of precise diagnosis and interpretation of mild or complicated chromosomal abnormalities [9]. These procedures yield information that serves as a basis for determining the appropriate clinical therapy and genetic counseling for patients in need of such services [10].

Male infertility has long been linked to cytogenetic abnormalities, and chromosomal abnormalities are more common in azoo-and/or oligozoospermic male populations than in the general population [11]. Translocation anomalies may have an inverse relationship with sperm motility, however, normal hormone levels, testicular volume, and sperm count are severely impacted by Y chromosome microdeletions and sex chromosomal disorders such as Klinefelter's (47, XXY) [12].

The medical history of all patients was unremarkable. There was no family history of drug or environmental toxin exposure, nor congenital malformations. Routine analyses showed no infectious cause for their infertility. Patients in our study had low to normal testosterone levels and high to normal levels of FSH and LH. Semen analysis showed an abnormal profile. Semen abnormalities may result from multiple causes genetic causes.

One patient was heterozygous for CFTR (c.3209G>A) and had another mutation in the UBR2 gene (heterozygous for UBR2 c.1674+5G>T). Fertility is one of the organ systems affected by the hereditary disorder known as cystic fibrosis (CF). Some mutations in the gene encoding cystic fibrosis transmembrane conductance regulator (CFTR) can lead to congenital absence of the vas deferens (CAVD) as a monosymptomatic form of CF [13]. CAVD impedes sperm transport and can lead to infertility in men with CF [14]. In addition, according to Miyamoto et al. (2011), patients who experience meiotic stoppage due to azoospermia may also be at genetic risk for UBR2 gene-related disorders [15].

Another patient was diagnosed with Klinefelter syndrome. With an estimated prevalence of 1:500/1000 per male live birth, Klinefelter syndrome, which is caused by an extra X chromosome, is the most common chromosomal sexual aberration (karyotype 47, XXY) [16]. Symptoms include small testicles, small penis, gynecomastia, feminine body proportions and hair, high stature, visceral obesity, and testicular failure [17]. 47, XXY is typically the result of a random error in chromosome separation during the creation of sperm cells, which results in the development of sperm cells with an extra copy of the Y chromosome. It is generally not inherited [16].

In certain instances, nondisjunction during cell division during post-zygotic mitosis in the early stages of embryonic development leads to the insertion of an additional Y chromosome. This can create mosaics that are 46, XY/47, XYY [18]. While the extra Y chromosome is deleted during meiosis, men with a 47, XYY karyotype are typically fertile and show no signs of passing it on to their offspring. However, they are sometimes reported in infertile populations [10].

The third case presented cytogenetic changes; the patient had increased length of the heterochromatin of chromosome 9 with 46XY,9qh+ as shown in Figure 2. No other genetic abnormalities had been detected with DNA sequencing. Chromosome 9 is the most variable morphologically, with an estimated 6-8% of the general population experiencing an extension of the heterochromatin 9qh+ [19]. It has been proposed that heterochromatic variations, such as 9qh+, may affect the morphological differentiation that leads to synaptonemal complex formation and, as a result, meiotic arrest [19]. Moreover, studies showed that the fraction of malformed spermatozoa in the 9qh+ group was significantly larger than in the chromosomally normal group and speculated that heterochromatic variants like 9qh+ could be one of several unknown factors disturbing normal gametogenesis [19].

## Conclusions

In conclusion, this report emphasizes how crucial a genetic assessment is when a man is infertile. Translocations and other chromosomal abnormalities can have a substantial effect on fertility and raise the possibility of passing on genetic diseases to progeny. When it comes to idiopathic male infertility, genetic mutations are still another crucial aspect to consider. Couples seeking to lower the risk of passing on genetic diseases and produce a successful pregnancy may find that ART combined with PGT is a suitable option.
